# A Food Photograph Series for Identifying Portion Sizes of Culturally Specific Dishes in Rural Areas with High Incidence of Oesophageal Cancer

**DOI:** 10.3390/nu5083118

**Published:** 2013-08-06

**Authors:** Martani Lombard, Nelia Steyn, Hester-Mari Burger, Karen Charlton, Marjanne Senekal

**Affiliations:** 1 Division of Human Nutrition, Stellenbosch University, Cape Town 8000, South Africa; 2 Population Health, Health Systems and Innovation, Human Sciences Research Council, Cape Town 8000, South Africa; E-Mail: npsteyn@hsrc.ac.za; 3 Department of Biochemistry, Stellenbosch University, Cape Town 8000, South Africa; E-Mail: hester.burger@mrc.ac.za; 4 PROMEC Unit, South African Medical Research Council, Cape Town 8000, South Africa; 5 School of Health Sciences, Faculty of Health & Behavioural Sciences, University of Wollongong, NSW 2500, Australia; E-Mail: karenc@uow.edu.au; 6 Division of Human Nutrition, University of Cape Town, Cape Town 8000, South Africa; E-Mail: marjanne.senekal@uct.ac.za

**Keywords:** fumonisin, mycotoxins, oesophageal cancer, food photograph series, maize, dietary intake

## Abstract

Rural areas of the Eastern Cape (EC) Province, South Africa have a high incidence of squamous cell oesophageal cancer (OC) and exposure to mycotoxin fumonisin has been associated with increased OC risk. However, to assess exposure to fumonisin in Xhosas—having maize as a staple food—it is necessary to determine the amount of maize consumed per day. A maize-specific food frequency questionnaire (M-FFQ) has recently been developed. This study developed a food photograph (FP) series to improve portion size estimation of maize dishes. Two sets of photographs were developed to be used alongside the validated M-FFQ. The photographs were designed to assist quantification of intakes (portion size photographs) and to facilitate estimation of maize amounts in various combined dishes (ratio photographs) using data from 24 h recalls (*n* = 159), dishing-up sessions (*n* = 35), focus group discussions (FGD) (*n =* 56) and published literature. Five villages in two rural isiXhosa-speaking areas of the EC Province, known to have a high incidence of OC, were randomly selected. Women between the ages of 18–55 years were recruited by snowball sampling and invited to participate. The FP series comprised three portion size photographs (S, M, L) of 21 maize dishes and three ratio photographs of nine combined maize-based dishes. A culturally specific FP series was designed to improve portion size estimation when reporting dietary intake using a newly developed M-FFQ.

## 1. Introduction

A relatively small geographic area in rural areas of the Eastern Cape (EC) Province of South Africa has a high incidence of squamous cell oesophageal cancer (OC) [1]. The aetiology of OC is still unclear; however various risk factors have been associated with the disease including alcohol consumption [2], tobacco use [2], and exposure to the carcinogenic mycotoxin fumonisin [3]. Of particular interest in the South African context is the high exposure to mycotoxins (fumonisin moniliforme) in these areas [3,4,5]. Past research has shown that fumonisin grow on maize that is stored in suboptimal damp conditions and are found in higher concentrations in home-grown compared to commercially sold maize [5]. Maize is the primary staple food of the black population of South Africa and is consumed in large amounts on a daily basis in the geographical area of interest [3,4]. Fumonisin contamination of this food source is a major health concern and to date no quantitative assessment of exposure has been conducted [6]. Recently, a culturally specific maize-specific food frequency questionnaire (M-FFQ) was developed [7] to determine fumonisin exposure from maize consumption in rural-dwelling residents of the EC Province.

Accurate assessment of fumonisin exposure in isiXhosa-speaking subsistent farmers whose staple food is maize requires determination of maize consumed in various dishes throughout the day. Maize is typically consumed with vegetables, and the ratio of maize to vegetables varies according to availability of vegetables [8,9]. This variation to commonly consumed dishes makes it difficult to estimate the actual amount of maize consumed and requires determination of the ratio of maize to vegetable.

Minimizing measurement error is important when determining diet-disease association. Classification of people based on their food and therefore nutrient intake is determined by the accuracy of the dietary assessment tool used. Therefore, using dietary assessment tools with known measurement errors, the attenuating effects of misclassification can be assessed and interpreted [10].

A key error occurring in the measurement of food intake occurs during portion size assessment. The use of scales is often problematic or not appropriate and then the assessment of food intake depends on the participant’s ability to remember and describe their usual portion size [10]. Various aids have been developed to improve participants’ description of their usual portion size (such as portion size models, food models, photographs and schematic diagrams).

In a study conducted by Nelson *et al.*, (1996) it was concluded that photographs that depicts a range of portion sizes can be used to improve portion size estimation and that the use of such photographs can further reduce the misclassification of participants. It was however reported that factors such as participant age, gender and body mass index can influence portion size estimation [10].

Owing to culturally specific dietary habits [11], existing food portion size photographs that represent Western, mainly urban-type meals could not be used in this population. The aim of the present study was to develop a culturally specific series of food photographs (FPs) to be used as an aid when conducting dietary interviews in order to improve portion size estimation of maize-based meals and beverages, such as traditional maize-beer.

**Figure 1:**
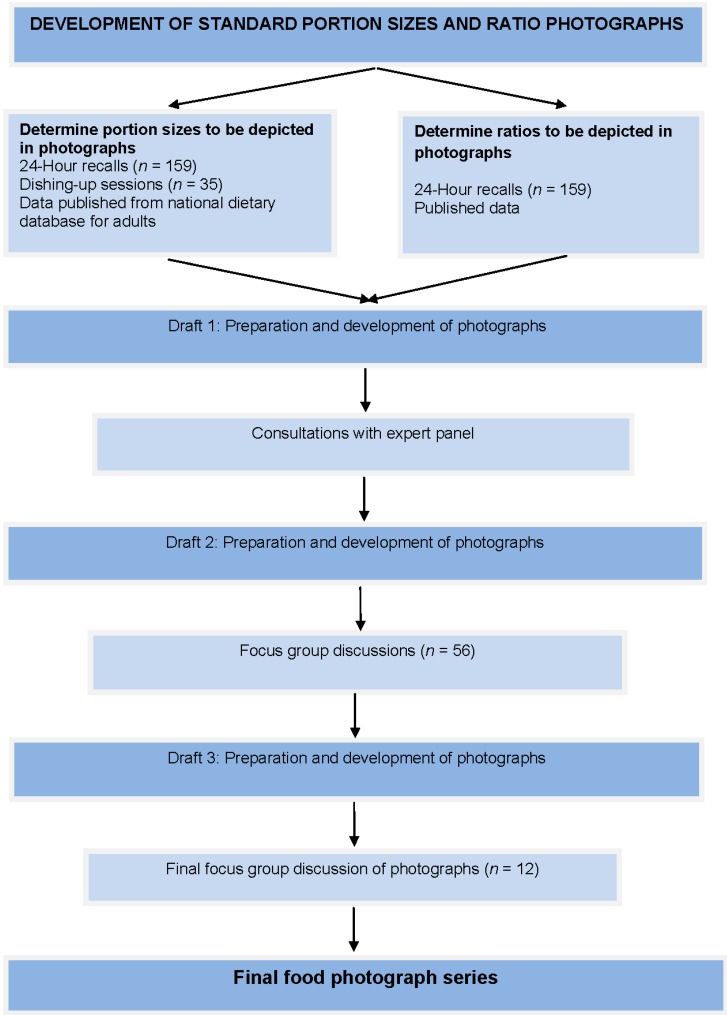
Process followed for the development of the portion size and ratio photographs.

## 2. Materials and Methods

### 2.1. Overview

As shown in Figure 1, the initial FPs on maize, maize-vegetable combined dishes, and maize beverages (*n* = 21) were developed based on data obtained from (i) a survey using 24 h recalls from EC Province; (ii) women from the study area dishing up their “usual” serving portions and (iii) data on portion sizes obtained from a national database. In addition to the determination of portion size it was also necessary to determine the ratio of maize to vegetables in “combined dishes”. This was done by reviewing published data and data from a study using 24 h recalls from the EC Province.

After the initial FPs were finalised (Draft 1) they were shown to an expert group for comment; the FPs were subsequently revised (Draft 2). This was followed by focus group discussions (FGD) with participants recruited from the target study population, who made further recommendations, which resulted in Draft 3. A last FGD resulted in the final set of FPs. Each step is discussed in detail below.

### 2.2. Determination of Portion Sizes

#### 2.2.1. Use of 24-h Recalls

Standard portion sizes of maize items, dishes and beverages appearing on the M-FFQ were determined using 24 h recall data (*n* = 159) collected in the same areas [7]. Participants provided information on the maize based food items and dishes consumed the previous day, as well as snacks and beverages to provide information on the dietary habits of the people living in these areas. The objective of the reviews was to identify commonly consumed food items and dishes as well as cooking and food preparation methods.

#### 2.2.2. Dishing up Sessions

Dishing-up (serving) sessions were undertaken in the selected areas to determine the standard portion sizes of these M-FFQ dishes: stiff *pap* (maize meal porridge with a stiff, thick consistency), soft porridge (maize meal porridge having a thin consistency), samp (whole maize kernels) and beans, spinach/*imifino* (a wild leafy vegetable similar to spinach) combined with *pap*, pumpkin combined with *pap*, and soup/*isophi* (watery soup consisting of whole maize kernels and dried sugar beans). Two rural areas of the EC Province were selected because of the high incidence of OC [1]. Because of poor infrastructure; villages from each area were randomly selected. Female volunteers were recruited with snowball sampling. Six women were identified and prepared the six most commonly consumed maize dishes using ingredients provided by the research team.

Thirty five female volunteers (18–55 years) were recruited and asked to dish up a “usual” portion that they would serve an adult male and female living in the household. The served portions were individually weighed on the plate. The weight of the plate subtracted to provide a portion size. Participants were informed that they would not be eating the food so that neither their current state of hunger, nor the fact that the food was free would influence the dishing up process [12].

The mean weight for each dish was determined and the inter quartile (IQ) range (25%, 50% and 75% percentiles) were used to determine S, M, and L portion sizes of each dish.

#### 2.2.3. Data from a National Database

Portion sizes for dishes not included in the dishing-up sessions were determined from a national dietary database on dietary intake compiled from studies that were conducted among different population groups in South Africa from 1983 to 2000 [13]. Two studies conducted in similar rural communities formed part of this data base.

### 2.3. Determination of Ratios Depicted in Food Photograph Series

Standard ratios of maize to vegetables in nine combined dishes (maize meal + *imifino*; maize meal + spinach; maize meal + pumpkin; maize meals + dried beans; maize meal + dried beans; samp + beans; *isophi*; mealie rice + spinach; mealie rice + pumpkin) were determined using previously unpublished 24 h recall data from our group (*n* = 159) [7], as well as review of previously published data [8,9].

### 2.4. Preparation and Development of Initial Photographs

Maize dishes depicted on the initial FPs were prepared by a woman born and raised in the area. Raw ingredients were weighed and cooking methods and preparation steps recorded. An initial FP series (Draft 1) was developed. Photographs were taken at an angle of 42° above the horizon, which is the average angle of viewing when a person is seated at a table) [14]. Different colour backgrounds, reference scales, types of and colour plates were considered in the process (Table 1).

**Table 1:** Different factors considered in developing the photographs.

Factors considered	Photograph options	Influence of photographs
Colour of the plate	White	Maize dishes are white and therefore there was little contrast between the food and the plate.
	Yellow or cream	Influenced the colour of dishes containing pumpkin.
	Green	Influenced the colour of dishes containing spinach.
	Blue	Influenced some of the photographs containing spinach.
Type of plate used	Bowl	Determining the depth of the dish was difficult when it is presented in a bowl.
	Plate	Determining depth on a plate was easier than that of a bowl.
Background of the photograph	Dark (navy or black)	White maize dishes were more pronounced on a dark background.
Scale	Match box	This was disregarded because smoking is a risk factor for cancer and would send mixed messages.
	Ruler	A ruler is not a known or much used item in this rural area.
	Knife and fork	These utensils are not frequently used in the area.

### 2.5. Evaluation of Draft 1 of the Food Photograph Series

An expert panel (consisting of two research dieticians and a research nutritionist experienced in the development of dietary assessment methods and the culture of isiXhosa speaking people) evaluated Draft 1 of the FP series. A second set (Draft 2) of FPs was subsequently developed to address the comments by the expert panel. 

### 2.6. Evaluation of Draft 2 of the Food Photograph Series

Draft 2 of the FP series was evaluated by means of FGD in the area. Xhosa women aged 18–55 years who were born and raised in the area were invited to participate in the FGD (*n* = 56). Men were excluded from the FGD since food preparation is regarded as “women’s business” in these areas [7,8,9].

Two nurses from the area were trained to facilitate the FGD using a structured interview guide prepared by the research team. Two local women, who were identified by the facilitators to host the discussions, were provided with information regarding the number and age of the required participants. The women invited participants according to the inclusion criteria.

Participants critically reviewed and discussed whether Draft 2 of the FP series truly reflected the various traditional dishes and portion sizes consumed by adults in the area. All discussions were audio-recorded, transcribed and translated to English by an isiXhosa speaking interviewer. A third draft of FP series (Draft 3) was developed to address the relevant comments raised by the FGD participants.

### 2.7. Evaluation of Draft 3 of the Food Photograph Series

A final FGD was conducted with 12 women aged 18–55 years who had been raised in the area. The participants critically reviewed and discussed the applicability and recognisability of these photographs to the target population and also identified the three most recognisable and commonly consumed ratio photographs. The appropriateness of the actual sizes of the photos was also discussed.

Information obtained from the various methods described above was integrated to provide a final FP series to accompany the newly developed M-FFQ [7].

### 2.8. Ethics

Ethical approval for the study was obtained from the Research Ethics Committees of the University of Cape Town (UCT) (123/2003) and the Medical Research Council (MRC) of South Africa. Each participant received detailed, easy to understand information (both verbally and written) regarding the study, and written consent was obtained in the participant’s first language (isiXhosa).

## 3. Results

### 3.1. Socio-Demographic Description of Participants in the Quantitative and Qualitative Assessments

In summary, mean age of the participants was 44 years (±16), most lived in traditional mud houses, used river water as primary water source and had either no, or limited schooling (Table 2).

**Table 2:** Socio-demographic description of participants.

Socio-demographic characteristic	24 h recalls *n* = 159	Dishing up session *n* = 35	Focus group discussions *n* = 56	Final Focus group discussion *n* = 12
	*n* (%)	*n* (%)	*n* (%)	*n* (%)
**Education**				
No formal education	69 (43)	6 (17)	14 (25)	1 (8)
Primary school (grade 1–7)	60 (38)	15 (43)	15 (27)	3 (25)
Secondary school (grade 8–12)	30 (19)	14 (40)	27 (48)	8 (67)
**Employment**				
Unemployed	87 (55)	25 (71)	28 (50)	4 (33)
Employed	72 (45)	10 (29)	28 (50)	8 (67)
**Monthly income**				
R 500–R 1000 (63–126 USD)	142 (89)	23 (66)	49 (87)	5 (42)
>R 1000 (126 USD)	17 (11)	12 (34)	7 (13)	7 (58)
**No. of people financially contributing to the household**				
1 Person	133 (84)	21 (60)	52 (78)	7 (58)
2 Persons	26 (16)	14 (40)	4 (7)	5 (42)
**Type of housing**				
Traditional mud houses	144 (91)	19 (54)	40 (67)	0 (0)
Brick houses	15 (9)	16 (46)	16 (28)	3 (25)
Informal structures	0 (0)	0 (0)	0 (0)	9 (75)
**Water source**				
River water	137 (86)	19 (54)	34 (62)	0 (0)
Communal tap	22 (14)	16 (46)	15 (27)	8 (67)
Inside tap	0 (0)	0 (0)	7 (12)	4 (33)
**Fuel for cooking**				
Fire	140 (88)	21 (60)	35 (63)	5 (42)
Electricity	19 (12)	14 (40)	21 (37)	7 (48)

### 3.2. Portion Sizes and Ratios Determined by Various Methods

Table 3 shows that portion sizes of maize dishes derived from the 24 h recalls ranged from 490 g (porridge) to 771 g (stiff *pap* and pumpkin). Results from the dishing up sessions are presented in Table 4. Soft porridge had the smallest portion sizes, and samp and beans the largest. Data on ratios are presented in Table 5.

**Table 3:** Portion sizes derived from 24-h recalls conducted in the Eastern Cape Province (*n* = 159).

Dish	Percentiles			Minimum	Maximum	Mean	SD
*n* = 159	25% (Small)	50%	75% (Large)				
Maize porridge (g)	338	470	590	184	862	490	176
Stiff pap * (g)	375	501	703	210	1290	555	234
Stiff *pap* and cabbage (g)	364	526	707	255	930	547	193
Stiff *pap* and pumpkin (g)	656	727	824	524	1250	771	227
Stiff *pap* and imifino ** (g)	448	588	770	330	985	600	204
Stiff *pap* and spinach (g)	328	426	530	310	1170	531	323
Stiff *pap* and beans (g)	445	544	765	376	935	613	232
Samp and beans (g)	328	468	646	150	1290	536	261
Amagewu *** (mL)	300	450	1000	200	1000	600	355

50% medium = median; SD = standard deviation; * *Pap* = Porridge; ** *Imfino* = Wild green leafy vegetable; *** *Amagewu* = Maize meal beverage.

**Table 4:** Portion sizes of six main maize dishes calculated during the dishing-up sessions (*n* = 35) *.

Dish	Inter quartile percentiles			Minimum	Maximum	Mean	SD
*n* = 60	25% (Small)	50%	75% (Large)				
Porridge (g)	358	557	630	146	760	497	174
Stiff pap ** (g)	308	478	640	164	1204	505	232
Samp and beans (g)	592	744	896	294	1396	743	217
Stiff *pap* and pumpkin (g)	462	592	794	196	1201	626	237
Stiff *pap* and imifino *** (g)	426	512	710	102	1303	561	227
Soup (g)	466	608	726	228	916	587	177

50% medium = Median; SD = standard deviation; * Female volunteers were asked to dish up a portion for herself and a portion for a man/husband (5 women did not have a man/husband thus dished up only one portion; ** *Pap* = Porridge; *** *Imfino* = Wild green leafy vegetable.

**Table 5:** Ratios depicted on the photographs.

Food type	Ratios		
	Cooked maize: Vegetable	Cooked maize: Vegetable	Cooked maize: Vegetable
Stiff pap + *imifino*	1:1	1:2	2:1
Stiff pap * + spinach	1:1	1:2	2:1
Stiff pap * + pumpkin	1:3	3:1	1:2
Stiff pap * + dried beans	1:2	2:1	1:3
Samp and beans	2:1	3:1	5:1
Soup	1:1	2:1	1:2
Mealie rice + *imifino*	1:3	3:1	1:2
Mealie rice + spinach	1:3	3:1	1:2
Mealie rice + pumpkin	1:3	3:1	1:2

* *Imfino* = Wild green leafy vegetable; ** Pap = Porridge.

### 3.3. Feedback Processes Leading to Development of Final Photographs

The initial photographs were handheld size (10 × 15 cm) and were shot against a black background, on a white plate with a ruler for scale The expert group decided that the black background was too dark, that the ruler was not a familiar item in the area studied, and that the use of white plates made it difficult to determine portion sizes of the mostly white food items.

Draft 2 of the FP series were taken against a white background with more familiar utensils to illustrate scale (fork, knife and spoon). The food items, prepared by an isiXhosa speaking woman from the area, were dished up onto a light blue enamel plate which is commonly used in households in the area and hence familiar to the participants. Portion size photographs of the traditional beer and other maize based beverages were represented in similar blue enamel mugs. Actual size of the photographs remained the same (10 × 15 cm).

During the FGD, participants commented that the hand-held photographs were too small to identify different portion sizes and that larger photographs were needed. They further commented that it was difficult to see and to determine depth of the portion sizes, especially the white maize dishes on a white background. They also mentioned that the fork, knife and spoon depicted were not acceptable as a scale for indicating dimensions and had to be replaced.

**Figure 2:**
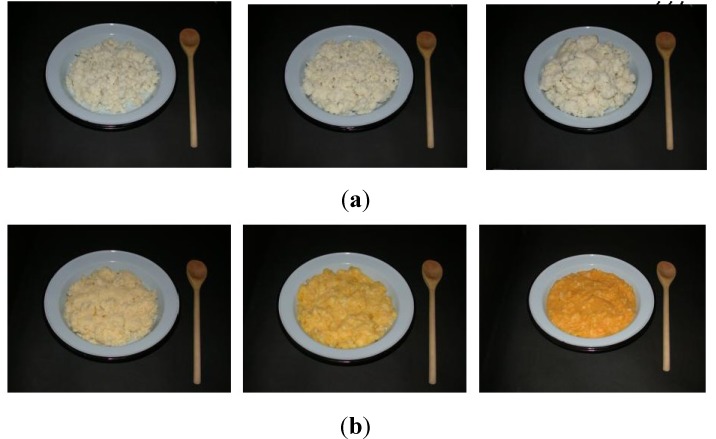
(**a**) Draft 3, portion size photographs for stiff *pap*; (**b**) Draft 3, ratio photographs for maize meal and pumpkin.

A third draft (Draft 3) of photos was taken using a black background to emphasise the mostly white foods. A light blue plate was used with a wooden spoon (most common dishing up utensil) as a scale (Figure 2). All utensils used in the photographs were procured in the EC Province. Actual size of photographs was increased to 42 × 30 cm to provide life-size images of the dishes, since participants in the FGD concluded that life size photographs would be the easiest to correctly identify portion sizes.

Focus group discussion participants found it difficult to estimate the three-dimensional perspective of the brown bread, cabbage, spinach and stiff *pap* from the photographs. This was resolved by reducing the camera angle to allow more depth [14]. Participants also did not recognise the difference between the medium and large portions of brown bread and the combined cabbage and spinach dish. These food items and dishes were repositioned on the plate and re-taken. Lastly, FGD participants indicated that the depicted portion sizes of the traditional beer were too small and suggested it be replaced with larger portion sizes, in plastic jugs.

### 3.4. Final Food Photograph Series

The final FP series comprised three portion size photographs (S, M, L) of 21 maize food items (Figure 2a), dishes and beverages, including baked maize bread, steamed maize bread, *dumplings*, *vetkoek*, maize on the cob, whole kernels, soft porridge, stiff porridge, crumbly porridge, maize meal cooked with *imifino*, maize meal cooked with spinach, maize meal cooked with pumpkin, maize meal cooked with dried sugar beans, samp and dried sugar beans, soup (maize kernels and dried sugar beans), maize rice cooked with *imifino*, maize rice cooked with spinach, maize rice cooked with pumpkin, *amagewu* (maize beverage) and traditional maize beer.

The FP series also included three ratio photographs of the nine combined dishes (Figure 2b), including maize meal cooked with *imifino*, maize meal cooked with spinach, maize meal cooked with pumpkin, maize meal cooked with dried sugar beans, samp and dried sugar beans, soup (maize kernels and dried sugar beans), maize rice cooked with *imifino*, maize rice cooked with spinach, maize rice cooked with pumpkin.

Coding on the reverse side of the photographs correspond with that on the M-FFQ in order to shorten the dietary interview process and allow easy identification of the relevant portion size of a food that was consumed within the reference reporting period.

## 4. Discussion

The primary aim of the present study was to develop a FP series to improve portion size estimation when using a newly developed maize-based FFQ (M-FFQ). Both the FP and the M-FFQ were developed specifically for use in rural dwelling South Africans who are exposed to fumonisins through maize consumption [7]. A systematic approach was used to identify culturally appropriate serving sizes for the food items, dishes and beverages, and FPs were taken using these portions.

Measurement error in dietary assessment usually occurs because participants are unable to describe portion sizes accurately [10]. Predetermined portion sizes on FFQs simplify the coding and data entry process and are therefore useful in epidemiological surveys that include large numbers of participants [15]. Due to large potential between-person and within-person variation, particularly seasonally [15], the approach was to include three portion sizes for the most commonly consumed maize-based food items included in the M-FFQ. Most rural people, many of whom have low literacy levels, find it easier to identify portion sizes from local measures (cups, glasses, bundles, heaps or numbers) rather than from measuring units [16]. Two-dimensional models such as photographs and food models have been shown to increase accuracy during portion size estimation in illiterate populations [17].

A strength of the study was the use of qualitative methods, both in-depth interviews and FGD with key informants, to develop photographs as an aid to use with a quantitative dietary assessment tool [18]. Culturally-specific food preparation techniques and recipes were discussed in the FGD, and were confirmed during actual dishing-up (serving) sessions, in which the ingredients were weighed and preparation steps were recorded.

Careful consideration was given to ensure that the final food photograph series was depicted in an easy-to-identify layout with familiar utensils. In this regard, life-size photographs were found to be more effective than the smaller hand-held cards. This participatory research project involved support from local community representatives, recruited from the geographical area of interest. It is therefore envisioned that this culturally specific FPs will improve portion size estimation to ultimately provide more accurate information on the dietary habits and nutrient intakes of those living in rural areas in the EC Province of South Africa. However, it is of the utmost importance that the food photographs be validated to determine the presence and direction of bias in terms of portion size recalls of these participants [19]. Furthermore, the effect of only three portion size choices needs to be investigated as it is possible that more photograph choices (5–8) would decrease portion size estimation error [19,20]. These additional photographs should ideally represent intermediate values between the current S, M and L to further obtain personalized values.

## 5. Conclusions

A FP series to improve portion size estimation of people living in rural areas of the EC Province was successfully developed using a comprehensive process of accruing qualitative and quantitative information and expert opinions, as well as very close engagement with the target community.
